# Questionnaire for Orchestra Musicians: Validation of the Online Version of the Musculoskeletal Pain Intensity and Interference Questionnaire for Polish Musicians (MPIIQM-P)

**DOI:** 10.3390/jcm13061626

**Published:** 2024-03-12

**Authors:** Anna Katarzyna Cygańska, Michał Kaczorowski

**Affiliations:** Faculty of Rehabilitation, Jozef Pilsudski University of Physical Education in Warsaw, Marymoncka 34, 00-968 Warsaw, Poland; anna.cyganska@awf.edu.pl

**Keywords:** validation, MPIIQM, questionnaires, musicians

## Abstract

**Background**: The only complete and validated tool for evaluating professional orchestra musicians is the English-language Musculoskeletal Pain Intensity and Interference Questionnaire for professional orchestra Musicians (MPIIQM) questionnaire, which, in recent years, has been translated, adapted, and validated in other languages. The aim of the study was to validate the online version of the Polish version of the Musculoskeletal Pain Intensity and Interference Questionnaire for Musicians (MPIIQM-P). **Materials and Methods**: The group included 182 professional musicians. The respondents were asked to complete the MPIIQM-P, BPI, and QuickDash questionnaires twice within an interval of 4 days. The questionnaires were created in the web form creator—Google Form. **Results**: The EFA analysis showed a two-factor structure of the questionnaire consisting of factor 1—pain intensity and factor 2—pain interference. The internal agreement of the factors identified in the EFA analysis was measured by the α Cronbach index reaching from 0.813 to 0.913. The intraclass correlation coefficient of both factors ranged from 0.276 to 0.583. The analysis of convergent and divergent validity between the subscales of the questionnaire value ranged from 0.414 to 0.925. **Conclusions**: The online version of the MPIIQM-P questionnaire is a valid and reliable tool for the assessment of musculoskeletal pain and interference. The online MPIIQM-P questionnaire maintains the psychometric properties previously defined for the paper version.

## 1. Introduction

With the prevalence of musculoskeletal complaints in professional orchestral musicians and the specificity of work in this professional group, there is a need to develop a reliable tool for assessing the musculoskeletal system of musicians and the impact of the complaints on their quality of life. Currently, there are several paper questionnaires for assessing this professional group. The most commonly used and described in the literature are the Musculoskeletal Load and Physical Health Questionnaire for Musicians [1,2], the Musculoskeletal Pain Questionnaire for Musicians (MPQM) [3], and the Musculoskeletal Pain Intensity and Interference Questionnaire for Musicians (MPIIQM) [4].

However, the psychometric characteristics of the Musculoskeletal Load and Physical Health Questionnaire for Musicians have not been evaluated to date. Consequently, it is not a valid and reliable tool for evaluating the musculoskeletal system. Furthermore, the questionnaire takes 25 min to complete, thus limiting its practical application by lower response rates from the study group [1,2,5]. The MPQM questionnaire is a validated tool with specific psychometric characteristics, but it does not provide information on pain location and the impact of pain on psychosocial variables [3]. Currently, the MPIIQM is the most widely used tool in the scientific literature for assessing the musculoskeletal system of professional orchestral musicians. The MPIIQM questionnaire is a complete tool with specific psychometric properties, validated based on COSMIN standards [6,7]. Translation and cultural adaptation of the MPIIQM questionnaire has already been performed in German [8], Brazilian Portuguese [9], Polish [10], Portuguese [11], Turkish [12] and French [13].

To make it as easy as possible to collect information about patients’ perceptions of their health and treatment, it is possible to create online questionnaires or adapt those originally constructed in paper form into online form. Online questionnaires generate less cost and allow for reaching a larger group of respondents [14]. Furthermore, the processing of data collected online is less time-consuming [15]. In adapting or constructing a questionnaire in online form, it is important to take into account the technical limitations that can make it impossible, among other things, to adapt the questionnaire items in an unaltered form [14]. Currently, there are many questionnaires originally constructed in paper form that have been successfully adapted and validated in online form [16,17,18].

Developed to assess the musculoskeletal system of professional orchestral musicians, the MPIIQM questionnaire was also originally constructed in paper form and was successfully adapted into online form in Portugal by Zão et al. [11]. Validation of an online form is currently underway in Turkey [12]. In Poland, this tool has only been validated and adapted in paper form [10]. Currently, there is no validated online tool for evaluating professional orchestral musicians. The aim of the present publication was to adapt and validate an online Polish version of the Musculoskeletal Pain Intensity and Interference Questionnaire for Musicians (MPIIQM-P) questionnaire.

## 2. Material and Methods

The type of research design of the study was a cross-sectional study. The study group of 182 professional musicians (age 37.19 ± 9.62) consisted of 99 (55%) women and 83 (45%) men. The survey was conducted among professional orchestral musicians playing in professional symphony orchestras. Fifteen symphony orchestras from across Poland were invited to participate in the survey. Willingness to participate in the survey was declared by 10 of them. At the time of the survey, there were 848 professional musicians playing in the orchestras included in the survey (information confirmed by the administrations of the respective entities). Responses were returned by 182 musicians (21% response rate). Of the 182 respondents, 85 answered affirmatively to items 11 and/or 12, thus continuing with the further part of the MPIIQM-P questionnaire. The questionnaires were completed twice by 57 respondents (among them, 30 musicians completed the questionnaire to the end). All participants were informed that participation in the study was voluntary and anonymous, and gave informed consent to participate in the experiment.

The inclusion criteria for the study were: age over 18, playing in a professional orchestra, and Polish as a mother tongue. Amateurs who played an instrument or played in amateur orchestras were excluded from the survey.

The study received a favorable opinion from the Senate’s Research Bioethics Committee (SKE 01-01/2023) and was conducted in accordance with the guidelines of the Declaration of Helsinki [19]. The study was registered at www.ClinicalTrials.gov, identification number: NCT05903300.

The survey consisted of completing the online version of the BPI questionnaire, QuickDash, and the validated MPIIQM-P questionnaire twice. Respondents were asked to complete the questionnaires 4 days apart.

The MPIIQM-P questionnaire consists of 22 items and provides the following data: demographic data (items 1 and 2), job-related data (items 3 through 8), frequency of pain over time (items 9 through 12), pain location (items 13 and 14), pain intensity (items 15 through 18), and pain interference (items 19 through 23). Pain intensity is determined using a numerical scale on which the extreme values are 0 (“no pain”) and 10 (the most severe pain). Pain interference was also measured on an 11-degree numerical scale with the same numerical range, and the extremes in items 19 and 20 are described as 0 (“did not interfere”) and 10 (“completely interfered”), and in items 21 through 23, 0 (“no difficulty”) and 10 (“unable”). The overall level that determines pain intensity (max. 40 points) and pain interference (max. 50 points) are calculated based on the sum of the values obtained for each item, and the total score of the questionnaire is 90 points.

The Polish-language QuickDash questionnaire is a short version of the DASH upper limb disability questionnaire [20]. The questionnaire also contains additional modules, one of which is “Playing the Instrument” used in the survey. The module consists of 4 items on the interference of pain with playing the instrument. Rating on a scale of 1 (“no difficulty”) to 5 (“unable”). The total score is calculated by adding up the partial scores for each item and then the result is divided by 4, followed by subtracting 1, and eventually multiplied by 25. The score ranges from 0 to 100 points—the higher the score, the greater the pain interference with activities performed with the upper limb.

Brief Pain Inventory is a validated tool for assessing pain in patients with pain complaints [21]. It provides information on: the presence or absence of pain (item 1), pain location (item 2), pain intensity (items 3 through 6), ways of coping with pain and their results (items number 7 and 8), and pain interference with various aspects of life (item 9). Pain intensity is determined using a numerical scale on which the extreme values are 0 (“no pain”) and 10 (“pain as bad as you can imagine”). Pain interference was also measured on an 11-degree numerical scale with the same numerical range, and the extreme values are described as 0 (“did not interfere”) and 10 (“completely interfered”). The overall pain intensity (max. 10 points) is determined by adding up the scores obtained in items 3 through 6 and dividing the total by 4. The overall level of pain interference (max. 10 pts) is determined by adding up the answers obtained in item 9 and dividing the resulting value by 7. The higher the score, the greater the pain interference and pain intensity.

The process of validating the online version of the MPIIQM-P questionnaire consisted of the following stages: adaptation of the paper questionnaire into an online version, pilot study, data collection, and assessment of the psychometric characteristics of the validated questionnaire. The MPIIQM-P questionnaire and two other questionnaires, also in online form, were emailed to musicians by the administrative divisions of each institution. Emails requesting questionnaires were sent twice, 4 days apart, to allow for reliability analysis at two time points. The data were collected from April to May 2023. The questionnaires were created in the online form creator (Google Forms). The items were transferred to the online form unchanged. A few items were adapted due to the technical limitations of online forms.

**Figure 1:**
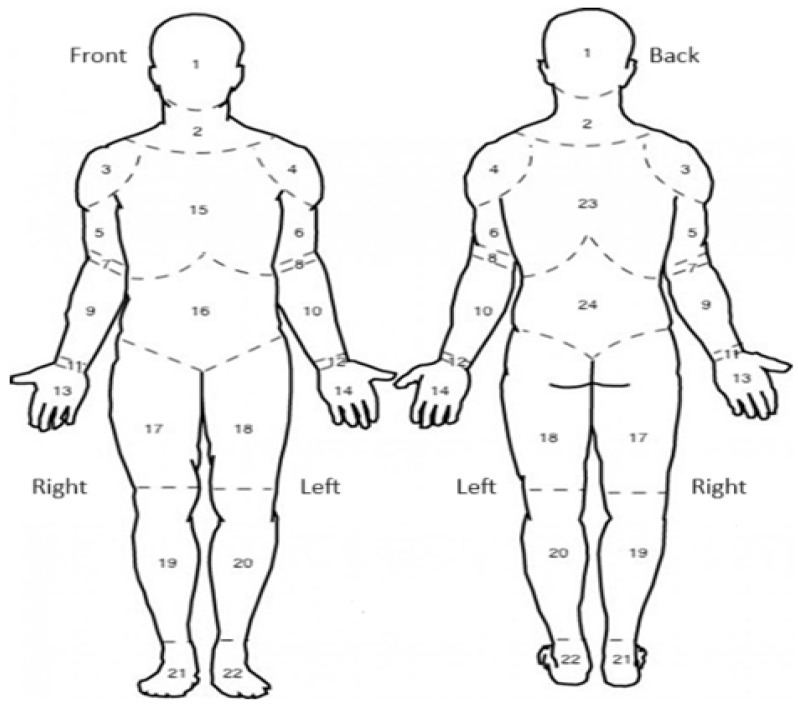
Adaptation of the body chart (question 13 of the MPIIQM-P) for online application.

In the online version of the MPIIQM-P questionnaire, answers to all questions were set as required. The respondent was unable to complete the questionnaire by skipping any question. For item 3: “What instrument do you play in the orchestra?”, instead of a short answer, a drop-down list of instruments was created from which the respondent made a choice. There was an option to select “Other” and input manually the instrument in the absence of a suitable one in the drop-down list. Item 13: “On the body diagram, please shade **each** area where you are experiencing pain/problem. Please **put an X in ONE area** that hurts the most.” has been adapted to “Based on the diagram below, **please choose the area of the body** that HURTS the most. Choose the side of the body (‘front’ or ‘back’), and in the question below, select the number corresponding to the area of the body you have chosen from the drop-down list.” The above change is due to technical limitations that prevent the creation of a body diagram in which the respondent could interfere. As a result of this change, the additional item 14 appeared: “Choose the number corresponding to the area of the body that HURTS the most” being a logical continuation of item 13. The consequence of this action is to change the original item numbers starting with item 15. The body diagram used in the online version of the questionnaire was appropriately adapted as recommended by the author of the original version of the questionnaire. The diagram used in the questionnaire is presented in Figure 1. This diagram was identical to that used by researchers preparing the Turkish version of the MPIIQM questionnaire [12]. The developed online form of the questionnaire in Polish is available at https://docs.google.com/forms/d/1phRHZNq8R2W1UEoAUC6bjT85z-M0zedNKtedcRVHToo (accessed on 23 January 2024).

As a result of the pilot survey conducted on a group of 10 professional musicians, an additional response form for item 4 was introduced in the MPIIQM-P questionnaire: “In what capacity are you employed in the orchestra?”. Originally, respondents had to give a one-choice answer by choosing between “full-time” and “half-time.” The “Other” option was added to provide a brief written response.

In the online version of the QuickDash questionnaire, a supplementary module “Playing the instrument” (the supplementary module also exists in the version for athletes) answers to all questions are set as required. The questionnaire’s request to specify in writing the instrument the respondent plays was changed to selecting an instrument from a drop-down list. There was an option to choose “Other” and input manually the instrument in the absence of a suitable one in the drop-down list. Furthermore, the form of items was adapted to the study group to avoid ambiguity. The sections on sports were removed because the study group consisted only of professional musicians. All items were adapted similarly to the example shown below. Item 1: “Have you had any difficulty applying your normal technique when playing the instrument or playing sports?” was replaced with: “Have you had any difficulty applying your usual technique when playing the instrument?”

In BPI’s online questionnaire, item 2: “Please circle the area where you are experiencing pain on the drawing. Please mark with an “X” the area where the pain is most severe” was adapted as: “Based on the diagram below, **please choose the area of the body** that HURTS the most. Choose the side of the body (‘front’ or ‘back’), and in the question below, select the number corresponding to the area of the body you have chosen from the drop-down list”, similar to the MPIIQM-P questionnaire. The original division of the body diagram was replaced by the diagram shown in Figure 1. The answer to the above item was not required because, in the case of a negative answer to items 11 and 12 of the MPIIQM-P questionnaire, the respondent not experiencing pain already answered this question. In item 8: “What treatments or medications do you receive for your pain?”, the written response was replaced with a single-choice answer. The respondent indicated the answer “I don’t receive any” or “I receive.” If the second option was selected, the respondent provided a written answer as to what treatment methods he or she used. In item 9, the percentage scale of relief (where 0% meant no relief and 100% meant total relief) was replaced by a numerical scale (where 0 meant no relief and 10 meant total relief) due to technical limitations preventing the original measure from being applied.

### Statistical Analysis

Statistical analysis using STATISTICA 13 software (TIBCO Software Inc., Palo Alto, CA, USA) was conducted to determine the following psychometric characteristics: convergent and divergent validity, reliability analysis in two time points, internal consistency, and exploratory factor analysis. Internal consistency was calculated using Cronbach’s α test reliability coefficient, while reliability analysis at two time points was performed using the intraclass correlation coefficient (ICC). An exploratory factor analysis (EFA) was conducted based on 9 items of the MPIIQM-P questionnaire (4 related to pain intensity and 5 to pain-related difficulties). The appropriateness of the use of EFA was confirmed by the Kaiser–Meyer–Olkin test prior to EFA. Convergent and divergent validity was calculated using the correlation coefficient for items providing information about pain and its interference from the MPIIQM-P, QuickDash, and BPI questionnaires. Quantitative data are presented using arithmetic means and standard deviations. Qualitative data are presented in percentage terms. The normality of the distribution of variables was verified using the Shapiro–Wilk test. The level of statistical significance was set at *p* < 0.05.

## 3. Results

Exploratory factor analysis (EFA) was calculated for participants who reported musculoskeletal pain associated with playing the instrument (*n* = 85). The Kaiser–Meyer–Olkin coefficient was KMO = 0.849, and the significance level of Bartlett’s sphericity test was *p* < 0.001. Two factors were extracted while meeting the Kaiser criterion for the eigenvalues of the extracted factors to be greater than 1. The explained variance was 73%. The EFA extracted factor 1 (pain intensity), which included 3 items (least pain, moderate pain, pain right now), and factor 2 (pain interference), which included 6 items (worst pain, mood, enjoyment of life, using the usual technique of playing the instrument, playing the instrument because of an ailment, playing the instrument as well as one would like). EFA factor loadings for each variable are shown in Table 1. Values in bold indicate factor loadings greater than 0.6.

Internal consistency was measured using Cronbach’s α index for factor 1 (pain intensity) and was 0.813, whereas for factor 2 (pain interference) it was 0.913. Reliability analysis at two time points was conducted using ICC. The intraclass correlation coefficient was calculated for the 3 items of the pain intensity factor and was ICC = 0.583. For the 6 items of the pain interference factor, ICC was 0.276. The results of the internal consistency and reliability analysis at two time points are shown in Table 2.

Based on the analysis of convergent and divergent validity, there was a significant correlation between the subscales of the MPIIQM-P questionnaire (pain intensity, pain interference, and impact of pain on playing the instrument), expressed by the sum of the scores obtained on these scales, and the subscales of the QuickDash and BPI questionnaires, at 0.414–0.925 (moderate to very strong correlation). Pearson correlation coefficients for both the questionnaire scores and subscales are shown in Table 3 For all variables shown in Table 3, the correlation was significant at *p* < 0.01, except for those marked with *, for which the *p*-value was *p* < 0.05.

## 4. Discussion

The research discussed in the present paper was the first in Poland and one of the first in the world to validate the online form of the MPIIQM questionnaire. The collected group of respondents was the second largest group in the study in the validation of the MPIIQM questionnaire and the largest group of professional orchestral musicians in Poland assessed for musculoskeletal complaints related to playing the instrument, based on the validated tool. The online form of the MPIIQM questionnaire was shown to retain the psychometric characteristics of its paper form, and the study identified characteristics that were not identified in a study by Cygańska et al. [10].

The group of respondents was 182 people, the second largest group in the research in this field. Only researchers conducting validation of the MPIIQM questionnaire in Brazil collected a larger group of 273 people [9]. During a validation study of the MPIIQM questionnaire, the response rate ranged from 33.8% [8] to 73.6% [11]. The response rate of professional musicians in the present survey was 21%. It is likely that the lower response rate is due to the online form of the questionnaire, which has a significantly lower response rate compared to paper-based questionnaires [22,23].

Assessment of psychometric characteristics confirmed the two-factor structure of the MPIIQM-P questionnaire, consisting of the factors of pain intensity and pain interference. The variable “Most severe pain” showed a strong correlation with each factor, although the highest one of 0.61 was demonstrated for pain interference, making it the variable part of that factor. The result is in opposition to previous studies in which the variable “Most severe pain” was consistent within the group with the pain intensity factor [8,9,10,11]. The correlations for other variables were consistent with previous validations conducted in other countries, excluding the validation conducted by Cygańska et al. [10], in which the variable “Pain right now” explained the pain interference factor. The factor loadings of the variables after EFA ranged from 0.61 to 0.87. In other studies in this field, the loadings ranged from 0.69 to 0.97 in the study by Berque et al. [4], from 0.59 to 0.94 in the validation conducted by Möller et al. [8], from 0.67 to 0.90 in a study by Zão et al. [11], from 0.57 to 0.91 in validation by Kochem et al. [9], from 0.68 to 0.96 in the validation of the MPIIQM questionnaire conducted by Cygańska et al. [10], and from 0.59 to 0.94 in a validation of the French-language version of the questionnaire by Roos et al. [13].

Internal consistency measured using Cronbach’s α index was determined to be good at 0.813 for the pain intensity factor and very good for the pain interference factor, with an index of 0.913 [24]. The internal consistency indices obtained are close to the results obtained by the author of the original MPIIQM questionnaire (0.91) for both factors identified in the EFA [4].

In a study conducted by Zão et al. [11] Cronbach’s α was 0.89 for pain intensity and 0.87 for pain interference. Möller et al. [8] reported the coefficients of 0.83 and 0.87, respectively. Kochem et al. [9] obtained the values of 0.87 for pain intensity and 0.91 for pain interference. Cygańska et al. [10] found the internal consistency of the two factors to be 0.78 and 0.92, respectively. Roos et al. obtained the coefficient ranging from 0.84 to 0.89 [13]. Internal consistency as measured by Cronbach’s α index in the present study is consistent with previous analyses and confirms the internal consistency of the online version of the MPIIQM-P questionnaire.

Based on the analysis of the intraclass correlation coefficient, there was a weak consistency between the individual variables of the two factors (pain intensity and pain interference) measured at the two time points, at 0.276–0.583 (no consistency to weak consistency). The results obtained are different from previous studies conducted in this area. Zão et al. [11] demonstrated excellent consistency at 0.985–0.999. Möller et al. [8] obtained a consistency of 0.79–0.92 (good to excellent). Kochem et al. [9] reported values of 0.76 to 0.84, indicating good compliance. Roos et al. [13] did not conduct an analysis of the intraclass correlation coefficient due to an insufficient number of respondents (*n* = 11). The low level of consistency may result from performing the analysis in a small group of participants (*n* = 30).

Analysis of convergent and divergent validity showed a strong correlation (r = 0.925) of the “Pain Intensity” subscale of the MPIIQM-P questionnaire with the identical subscale of the BPI questionnaire. Furthermore, there was a moderate correlation with subscales measuring pain interference of the BPI (r = 0.650) and QuickDash (r = 0.414) questionnaires. The subscale that assessed pain interference in the validated questionnaire showed a strong correlation with the identical subscales of the BPI (r = 0.714) and QuickDash (r = 0.798), and a moderate correlation (r = 0.531) with the “Pain Intensity” subscale of the BPI questionnaire. The results are not fully consistent with previous research in this field. All previous validations of the MPIIQM questionnaire have found a strong correlation (r > 0.7) between all subscales included in the survey [8,9,11,13]. The exception is the validation conducted by Cygańska et al. [10], who did not determine this index. The demonstrated discrepancies in convergent and divergent validity may be due to the different methodologies used for validating the questionnaire in online form. A respondent with current musculoskeletal pain, after completing the MPIIQM-P questionnaire and indicating the most painful location, was asked about the pain again on the BPI questionnaire, although the second question is not preceded by a comment indicating that the question is about the same pain location with the most severe pain. As a consequence of this ambiguity, the respondent in the BPI questionnaire may indicate another pain location and complete a further part of the questionnaire for the second pain location. In such a case, the natural consequence will be a reduction in the significance of correlations between the subscales of the questionnaires described. Furthermore, the likelihood of such a situation is supported by the fact that up to 43.4% of musicians experiencing musculoskeletal complaints report more than 5 painful body parts [25].

The use of the questionnaire in assessing the playing-related musculoskeletal disorders (PRMD) complaints of orchestral musicians can be effectively done with the MPIIQM-P. Its utility can be in assessing the location of pain and the pain interference. Analysis of the results can be done collectively for the entire orchestra, for different instrument groups of orchestra musicians (indicating the most severely affected musicians) or analysis of the results on the incidence of PRMD according to demographic and playing-related factors. The usefulness of the questionnaire lies primarily in its ability to assess cut points values, which can be used by physiotherapists and musicians to determine whether the current level of discomfort requires consultation with a physiotherapist or medical doctor. In further research, the validated tool could be used to determine the aforementioned cut points.

The limitations of the survey are mainly due to the need to adapt the online form of the questionnaire, as a consequence of the modification of item 13. Therefore, the online MPIIQM-P questionnaire provides information only on the most painful part of the body. In its original paper form, the questionnaire also collects information on less painful locations of the body. Further research on the MPIIQM questionnaire should take into account the above limitations.

The present study is also characterized by strengths. First, an online form was developed, and its psychometric characteristics were successfully assessed. The validation process assessed psychometric characteristics that were not possible to assess when the paper version was tested due to an insufficient number of responses [10].

## 5. Conclusions

1. The online version of the MPIIQM-P questionnaire is a valid and reliable tool for assessing musculoskeletal pain and related difficulties.
2. The online MPIIQM-P questionnaire retains the psychometric properties previously demonstrated for the paper version.

## Figures and Tables

**Table 1:** Factor loadings for 9 items of the MPIIQM following exploratory factor analysis.

Item	Varimax	
	Factor 1: Pain Intensity	Factor 2: Pain Interference
Worst pain	0.56	0.61
Least pain	0.83	0.29
Average pain	0.87	0.13
Pain right now	0.81	0.12
Mood	0.25	0.81
Enjoyment of life	0.34	0.77
Using your usual technique	0.20	0.87
Playing because of symptoms	0.16	0.83
Playing as well as you like	0.06	0.87

**Table 2:** Internal consistency and test-retest reliability for the 9 items.

Item	M	SD	ICC	Cronbach’s α	Cronbach’s α If Deleted
Pain intensity	12.67	5.21	0.583	0.813	-
Least pain	1.56	1.55	0.333	-	0.706
Average pain	3.27	1.33	0.656	-	0.730
Pain right now	2.88	1.76	0.420	-	0.802
Pain interference	17.82	10.18	0.276	0.913	-
Worst pain	4.95	1.71	0.616	-	0.910
Mood	3.93	2.48	0.697	-	0.894
Enjoyment of life	2.00	2.28	0.807	-	0.896
Using your usual technique	4.06	2.35	0.828	-	0.896
Playing because of symptoms	2.72	2.27	0.853	-	0.886
Playing as well as you like	4.95	1.71	0.800	-	0.897

**Table 3:** Pearson correlation coefficients between items and subscales of the MPIIQM-P and items and subscales of the BPI. items and subscales of the QuickDASH.

Items	MPIIQM-P Pain Intensity	MPIIQM-P Pain Interference (Interference in Performance)
BPI (Pain intensity)	0.925	0.531 (0.496)
Worst pain	0.780	0.706 (0.698)
Least pain	0.748	0.325 (0.240) *
Average pain	0.861	0.462 (0.433)
Pain right now	0.723	0.241 * (0.238) *
BPI (Pain interference)	0.650	0.714 (0.638)
General activity	0.604	0.545 (0.437)
Mood	0.482	0.739 (0.672)
Walking ability	0.460	0.391 (0.313)
Normal work	0.479	0.688 (0.685)
Relation with other people	0.551	0.382 (0.382)
Sleep	0.449	0.423 (0.350)
Enjoyment of life	0.452	0.474 (0.447)
QuickDash (pain interference)	0.414	0.798 (0.832)
Using the usual technique	0.402	0.659 (0.697)
Playing because of symptoms	0.345	0.730 (0.744)
Playing as well as you like	0.347	0.670 (0.717)
Spending your usual time playing	0.292	0.624 (0.634)

* *p*-value *p* < 0.05.

## Data Availability

Available upon request. Proposals should be directed to michal.kaczorowski@awf.edu.pl.
